# Beta-arrestin inhibits CAMKKbeta-dependent AMPK activation downstream of protease-activated-receptor-2

**DOI:** 10.1186/1471-2091-11-36

**Published:** 2010-09-21

**Authors:** Ping Wang, Yong Jiang, Yinsheng Wang, John Y Shyy, Kathryn A DeFea

**Affiliations:** 1 Division of Biomedical Sciences University of California Riverside, California, USA; 2 Environmental Toxicology Graduate Program, University of California Riverside, California, USA; 3 Department of Chemistry, University of California Riverside, California, USA; 4 Cell, Molecular & Developmental Biology Graduate program, University of California Riverside California, USA

## Abstract

**Background:**

Proteinase-activated-receptor-2 (PAR_2_) is a seven transmembrane receptor that can activate two separate signaling arms: one through Gαq and Ca^2+ ^mobilization, and a second through recruitment of β-arrestin scaffolds. In some cases downstream targets of the Gαq/Ca^2+ ^signaling arm are directly inhibited by β-arrestins, while in other cases the two pathways are synergistic; thus β-arrestins act as molecular switches capable of modifying the signal generated by the receptor.

**Results:**

Here we demonstrate that PAR_2 _can activate adenosine monophosphate-activated protein kinase (AMPK), a key regulator of cellular energy balance, through Ca^2+^-dependent Kinase Kinase β (CAMKKβ), while inhibiting AMPK through interaction with β-arrestins. The ultimate outcome of PAR_2 _activation depended on the cell type studied; in cultured fibroblasts with low endogenous β-arrestins, PAR_2 _activated AMPK; however, in primary fat and liver, PAR_2 _only activated AMPK in β-arrestin-2^-/- ^mice. β-arrestin-2 could be co-immunoprecipitated with AMPK and CAMKKβ under baseline conditions from both cultured fibroblasts and primary fat, and its association with both proteins was increased by PAR_2 _activation. Addition of recombinant β-arrestin-2 to in vitro kinase assays directly inhibited phosphorylation of AMPK by CAMKKβ on Thr172.

**Conclusions:**

Studies have shown that decreased AMPK activity is associated with obesity and Type II Diabetes, while AMPK activity is increased with metabolically favorable conditions and cholesterol lowering drugs. These results suggest a role for β-arrestin in the inhibition of AMPK signaling, raising the possibility that β-arrestin-dependent PAR_2 _signaling may act as a molecular switch turning a positive signal to AMPK into an inhibitory one.

## Background

β-arrestins were first identified for their role in mediating G-protein-coupled receptor (GPCR) desensitization and internalization, and were later discovered to serve as signaling scaffolds mediating G-protein-independent signaling. In our previous studies we have shown that Proteinase-activated-receptor-2 (PAR_2_) can signal through two different pathways, one involving Gαq coupling and mobilization of intracellular Ca^2+ ^and another involving recruitment of various signaling proteins into a scaffolding complex with β-arrestins [1-4]. As PAR_2 _is reported to have both protective and pathogenic effects in a number of diseases, the dominance of one pathway over the other may direct the ultimate physiological response[5,6]. Upon activation of PAR_2 _and a number of other receptors, β-arrestins can associate with and differentially regulate the activity of various signaling proteins. For example, association with β-arrestins increases the activity cofilin and ERK1/2, while inhibiting the activity of PI3K [1-4,7]. Furthermore, studies on other receptors suggest that β-arrestins can both positively and negatively regulate additional enzymes including RhoA, phosphatase PP2A and NF-κB[8,9].

PAR_2 _is one of a family of four GPCRs activated by proteolytic cleavage of their N-termini, which exposes a tethered ligand that then auto-activates the receptors. Synthetic peptides corresponding to the tethered ligand for PAR-1, 2 or 4 will specifically activate them in the absence of proteinase[10,11]. Members of this GPCR family share a common mechanism of activation, but they are quite divergent in their downstream signaling pathways. For example, while PAR_1 _and PAR_2 _can couple to Gαq, PAR_2 _exhibits β-arrestin-dependent desensitization and internalization, while PAR_1 _uses β-arrestins only for desensitization. Downstream of PAR_2_, β-arrestins scaffold and activate ERK1/2, while inhibiting PI3K. In contrast, β-arrestins increase PAR_1_-stimulated PI3K activity and inhibit ERK1/2 activation[1,12,13]. Previous studies suggested that Gαq-coupled receptors, including PAR_1_, promote AMPK activity through a Gαq/CAMKKβ-dependent mechanism, making AMPK a logical metabolic target of PAR_2_[14]; however, the role of β-arrestins in AMPK signaling have never been investigated. A major goal of this study was to examine the possible role of β-arrestins in the regulation of AMPK downstream of PAR_2_.

AMPK is a heterotrimeric serine/threonine kinase activated in response to decreased AMP/ATP ratios [15-17], by classic signaling pathways that increase CAMKK or LKB-1 activity, and by drugs such as statins, metformin and thiazolidinediones[18-20]. While AMP directly activates AMPK by inducing a conformational change and by rendering it less susceptible to dephosphorylation by protein phosphatases 2A and C [21,22], AMPK is further activated by phosphorylation on its α subunit at Thr 172 by LKB-1 or Ca^2+^/calmodulin kinase-kinase β (CAMKK β) [19,23,24]. Activation of AMPK is associated with a number of beneficial metabolic effects, including phosphorylation and inhibition of the enzyme acetyl coenzyme A carboxylase 1 (ACC1) which is involved in fat synthesis. AMPK activity is tightly regulated within the cell and there are a number of pathological conditions associated with decreased AMPK activity. Most research has focused on the mechanisms by which it is activated downstream of different receptors; however, the possibility that receptors can send negative signals to AMPK has not been as well studied. Given the ability of PAR_2 _to promote two separate signaling pathways leading to events that might be considered protective and pathogenic from a metabolic standpoint, we investigated whether it is capable of regulating AMPK and asked whether both Ca^2+^-dependent and β-arrestin-dependent signaling pathways were involved.

## Results

### PAR_2 _promotes CAMKKβ-dependent AMPK activity in fibroblasts

**Figure 1:**
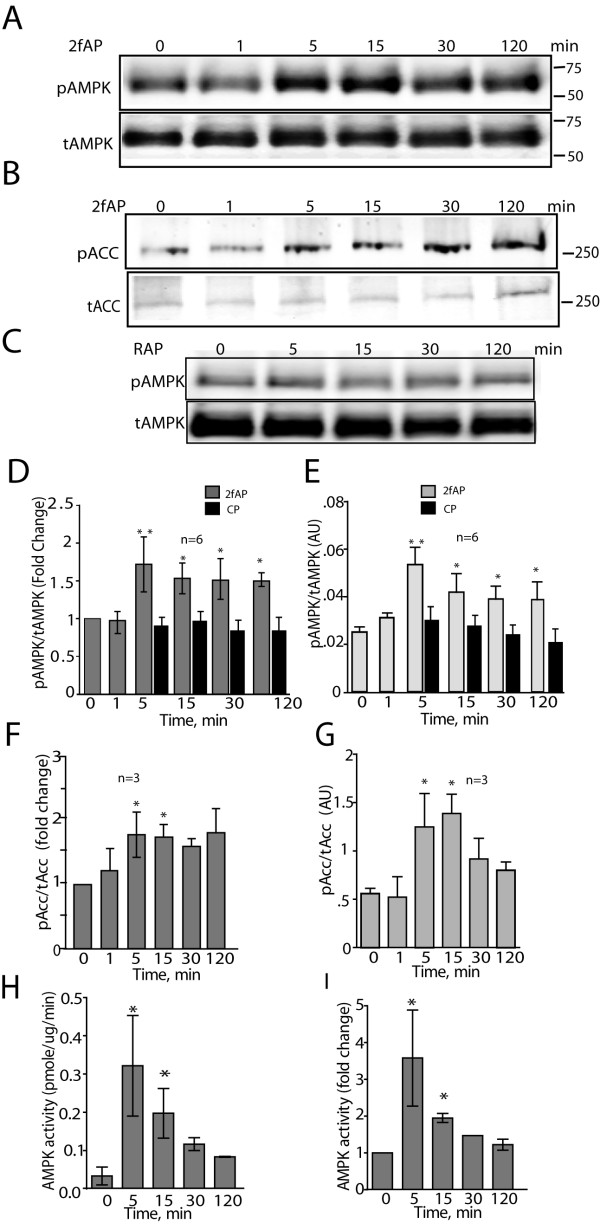
**PAR_2 _promotes AMPK activation in NIH3T3 cells**. **A-C**. Representative westerns of phospho- and total AMPK (**A, C**) or phospho- and total Acetyl CoA Carboxylase (pACC) (**B**) in lysates from cells treated with or without 100nM 2fAP (to activate PAR_2_) for 0-120 minutes (A, B) or treated with a negative control peptide containing the reverse tethered ligand sequence, 2-Furoyl-LRGIL-O (RAP) (C). Integrated band intensity was determined for phospho- and total AMPK and Acc using LICOR Odyssey software and phosphorylated protein was normalized to total protein levels for each sample. Graphs of fold changes over baseline (D, F) or raw values for normalized phospho-protein (E, G) are shown for AMPK (**D, E**) or Acc (**F, G**). Baseline is defined as pAMPK levels observed in the absence of 2fAP treatment. AU refers to integrated intensity values as arbitrary units. Statistically significant differences between treated and baseline are indicated by ** (p < .005) and * (p < .05). **G-H**. To determine AMPK activity, AMPK was immunoprecipitated from cells after treatment with 2fAP for 0-120 minutes, incubated with the substrate SAMS peptide in the presence of ^32^P-ATP and reactions spotted onto phospho-cellulose filters. Radiolabel incorporation was determined by scintillation counting of filters and nmoles of phosphate incorporated per mg of AMPK was calculated (**H**). Fold changes in AMPK activity were determined as the ratio of radiolabel incorporated in treated over untreated controls (**I**). (*statistically significant increase in activity, p < .05, n = 8).

To first determine whether PAR_2 _promotes AMPK activation, we treated NIH3T3 cells, with the PAR_2 _activating peptide 2-furoyl-LIGRL-O (2fAP) for 0-120 minutes and assessed AMPK phosphorylation by performing western blots with antibodies specific for Thr172 phosphorylated-AMPK and total AMPK (Figure 1A, C-D). A negative control peptide comprising the reverse sequence (2-furoyl-LRGIL-O) was used to show the response was specific to 2fAP. Although serine proteinases are the physiological activators of PAR_2_, synthetic peptide agonists corresponding to the tethered ligand are typically used to specifically activate the receptor, in an experimental setting, to minimize confusion from extraneous effects of proteinase treatment[11]. NIH3T3 cells were chosen for these initial studies because we have previously demonstrated that they favor Gαq over β-arrestin-dependent signaling pathways [2]. PAR_2 _promoted a 1.8-fold increase in AMPK phosphorylation, peaking at 5 minutes and remaining slightly elevated for 2 hours (Figure 1A). We simultaneously examined phosphorylation of a known substrate of AMPK, using an antibody specific for Ser79-phosphorylated ACC (Figure 1B, E-F), observing a similar increase in ACC phosphorylation with 2fAP treatment (Figure 1A-F). Reverse 2fAP did not increase AMPK phosphorylation, pointing to the specificity of the response (Figure 1C). To further confirm that the increase in AMPK phosphorylation reflected an increase in its activity, we immunoprecipitated AMPKα from cells after stimulation with 2fAP for 0-120 minutes and assayed phosphorylation of the AMPK substrate peptide (SAMS peptide); here we observed a 2-3-fold increase in AMPK activity that peaked at 5-15 minutes (Figure 1G, H). We conclude that PAR_2 _promotes phosphorylation and activation of AMPK, and its downstream substrate, ACC in NIH3T3 fibroblasts.

**Figure 2:**
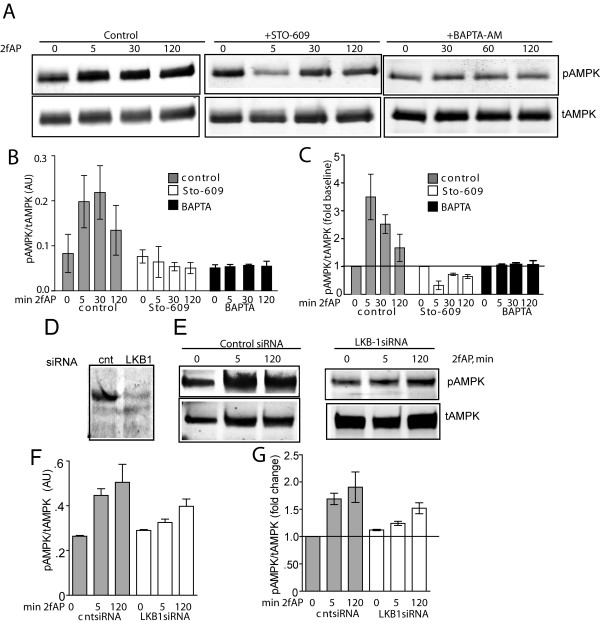
**PAR_2_-stimulated AMPK activation requires input from CAMKKβ and LKB-1**. **A**. Representative western blot of pAMPK and tAMPK in lysates from NIH3T3 cells, pretreated with vehicle (control, left panel), with 10 μg/ml STO-609 (middle panel), or with 300nM BAPTA-AM (right panel), followed by treatment with 2fAP for 0-120 minutes. Bar graphs showing raw normalized pAMPK levels (**B**) or fold changes over baseline in normalized pAMPK (**C**) (n = 4). Baseline is defined as pAMPK observed in the absence of 2fAP in vehicle-treated cells. **D-G**. Cells were transfected with control (cnt) or LKB-1 siRNA and AMPK phosphorylation was determined as described above. Representative westerns showing anti-LKB-1 with and without siRNA knockdown (**D**) and phospho and total AMPK levels with and without LKB knockdown (**E**) are shown. Bar graphs show normalized pAMPK levels (**F**) or fold changes in pAMPK relative to baseline (**G**) after cnt or LKB-1 siRNA (n = 3). Baseline is defined as pAMPK observed in cntsiRNA transfected, untreated cells.

PAR_2 _is a Gαq coupled receptor, which leads to mobilization of intracellular Ca^2+^. Since CAMKKβ is a Ca^2+^-regulated kinase that can be activated by PAR_2 _[25], and other Gαq-coupled receptors activate AMPK via CAMKKβ, we examined its role in PAR_2 _stimulated AMPK activity using the inhibitor STO-609. At the concentration used, (10 μg/ml), STO-609 is considered specific for CAMKKβ and although it exhibits non-specificity at higher concentrations, it does not affect the activity of LKB1, the other primary AMPKK [26]. In the presence of STO-609, PAR_2_-induded AMPK phosphorylation was blocked. In fact, although STO-609 treatment did not significantly decrease baseline pAMPK levels, we observed a mild decrease in AMPK phosphorylation below baseline levels upon PAR_2 _stimulation (Figure 2A-C). These data suggest that PAR_2 _is capable of inhibiting as well as promoting AMPK phosphorylation, an observation that is consistent with previous studies in which we demonstrated that a number of Gαq/Ca^2+^-dependent signaling pathways are opposed by β-arrestins and vice versa [2,4]. We conclude that PAR_2 _stimulated AMPK activation requires the activity of CAMKKβ and may be opposed by a separate PAR_2 _stimulated pathway. We address whether this inhibitory pathway is mediated by β-arrestins, similar to what has been observed for other proteins [2,4] in the next section.

**Figure 3:**
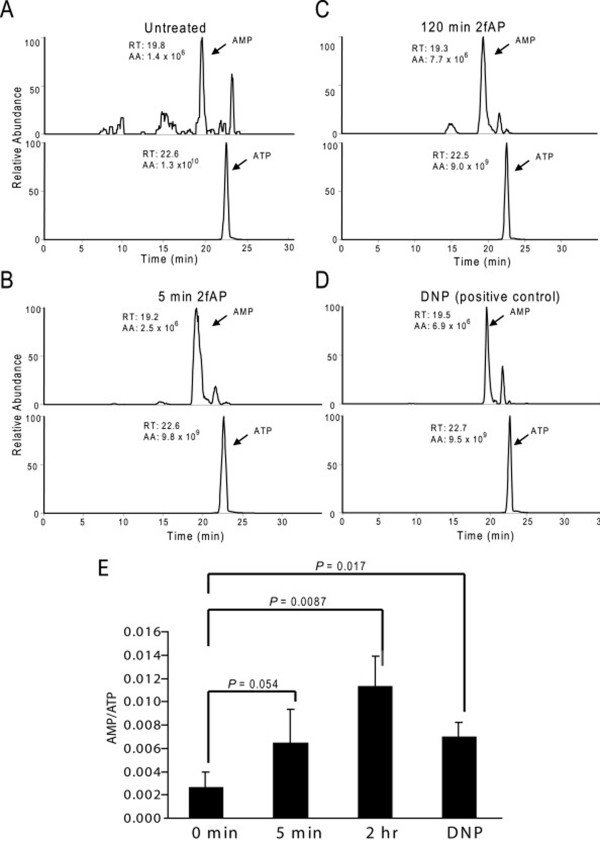
**PAR_2 _increases cellular AMP/ATP levels**. NIH3T3 cells were treated with 2fAP for 0-120 minutes or with DNP as a positive control. Nucleotides were extracted and analyzed by LC-ESI-MS/MS. **A-D**. Representative selected-ion chromatograms of ATP and AMP, shown above the peaks are the integrated peak area (AA) and retention time. **E**. Fold changes in AMP/ATP were calculated for each treatment group compared to untreated controls. The results were from three independent measurements.

The other kinase capable of activating AMPK is LKB-1, a tumor suppressor, which is activated by STRAD and STE-20-related kinases and which potentiates the effect of AMP on AMPK activity[27,28]. Transfection of siRNA to LKB-1 reduced LKB-1 protein by 70%, and resulted in a 50% decrease in PAR_2_-stimulated AMPK phosphorylation (Figure 2D-G). We next measured AMP and ATP levels in cells treated with or without 2fAP for 0-120 minutes by liquid chromatography-tandem mass spectrometry (LC-MS/MS). PAR_2 _increased AMP/ATP ratios at 120 minutes and to a lesser extent at 5 minutes (Figure 3). We conclude that LKB-1 also contributes to AMPK phosphorylation downstream of PAR_2_,, which may involve increased AMP/ATP ratios observed in response to PAR_2 _activation. Because CAMKKβ signaling downstream PAR_2 _is better understood, and the effect of CAMKKβ inhibition on PAR_2 _stimulated AMPK phosphorylation was more pronounced than that of LKB1, the remainder of these studies will focus on the CAMKKβ arm of this signaling pathway.

### β-arrestin-2 inhibits PAR_2 _stimulated AMPK activation

**Figure 4:**
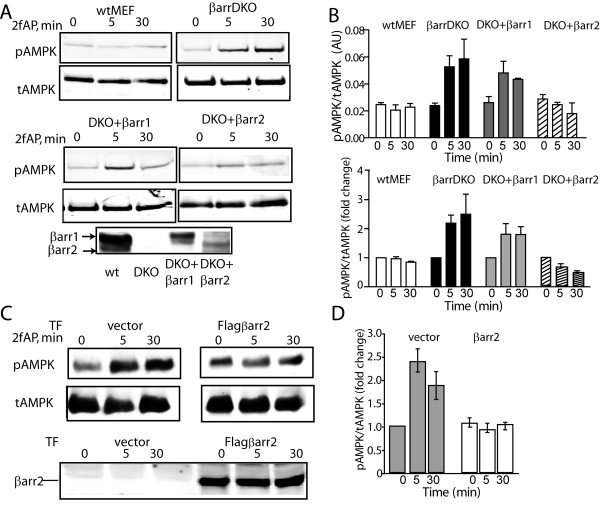
**Expression of β-arrestin-2 inhibits PAR_2 _stimulated AMPK activation**. A, B. Mouse embryonic fibroblasts from wt embryos (wtMEF), β-arrestin double knockout embryos (MEF βarrDKO), or MEFβarrDKO stably expressing βarrestin-1 (DKO+βarr1) or β-arrestin-2 (DKO+βarr2) were treated with 2fAP for 0, 5 or30 minutes and analyzed as described in Figure 1. **A**. Representative western blots of pAMPK and tAMPK in each cell line are shown in the upper two panels. The lower panel shows β-arrestin expression in each cell line. **B**. Bar graphs depicting normalized phospho-AMPK levels (upper) and fold changes over baseline in pAMPK/tAMPK (lower) in each cell line. Baseline is defined as pAMK observed in the absence of 2fAP for each cell line. **C**. Cells were transfected with an empty vector or flag-β-arrestin-2, treated with 2fAP for 0, 5 or 30 minutes and analyzed as described above. Upper panels: anti-pAMPK and tAMPK; lower panels: anti-β-arrestin was used to confirm expression of transgene. **D**. Bar graph showing fold changes compared with baseline in pAMPK in cells transfected with vector or Flagβarr2. Baseline in these experiments is defined as pAMPK in untreated, vector transfected cells.

In light of studies suggesting that PAR_2_-induced, Ca^2+^-dependent activation of other enzymes is inhibited by β-arrestins[2-4], we hypothesized that β-arrestins might be capable of inhibiting the PAR_2 _stimulated increase in AMPK phosphorylation. We examined AMPK phosphorylation in mouse embryonic fibroblasts from wild type mice (wtMEF), β-arrestin double knockout mice (MEFβarrDKO), or from MEFβarrDKO transfected with either β-arrestin-1 (DKO+βarr1) or β-arrestin-2 (DKO+βarr2). These transfected MEFs have been previously characterized and found to express levels of either β-arrestin-1 or 2 similar to those expressed in the wild type cells, and avoid the possible complications of compensatory mechanisms that may be present in either β-arrestin-1 or β-arrestin-2 knockout mice[29]. In wtMEF, no significant increase in AMPK phosphorylation was observed upon PAR_2 _activation, consistent with the higher levels of β-arrestins present in MEFs compared with NIH3T3 cells[2]. However, in MEFβarrDKO, and in MEFDKO+βarr1, PAR_2 _promoted a 2-2.5-fold increase in AMPK phosphorylation. In contrast, in MEFDKO+βarr2, PAR_2 _stimulated AMPK phosphorylation was inhibited (Figure 4A, B). Furthermore, when flag-β-arrestin-2 was over-expressed in NIH3T3 cells, PAR_2 _stimulated AMPK phosphorylation was abolished (Figure 4C, D). In NIH3T3 cells over-expressing β-arrestin-2, we observe a small (<10%) increase in baseline pAMPK (i.e. pAMPK levels in the absence of PAR_2 _agonists). We do not yet know the significance of this alteration in basal AMPK phosphorylation but may reflect a PAR-_2_-independent effect of β-arrestin on AMPK phosphorylation. β-arrestins are involved in terminating the signals of a number of receptors known to activate AMPK. PAR_2 _is relatively unusual in that it promotes a number of β-arrestin-dependent signaling events, while its G-protein signal is being dampened. We conclude that β-arrestins can inhibit PAR_2 _stimulated AMPK phosphorylation.

### PAR_2 _promotes β-arrestin-dependent inhibition of AMPK in primary fat

**Figure 5:**
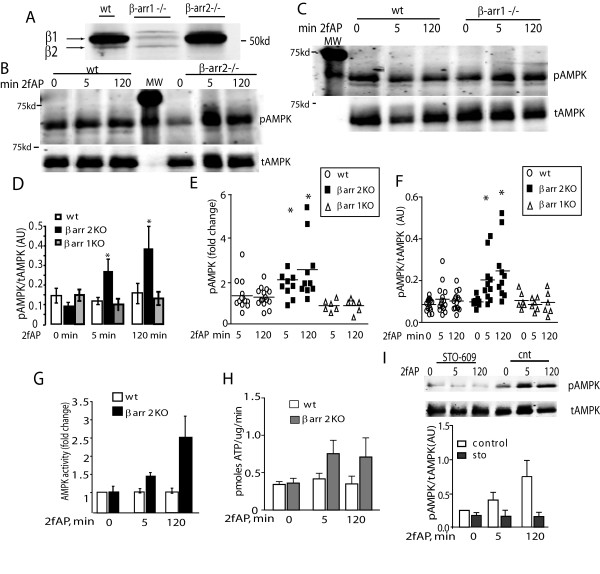
**PAR_2_-induced AMPK activation in primary fat is enhanced in the absence of β-arrestin-2**. A. β-arrestin expression levels in extracts from wt mice, β-arrestin-1^-/- ^mice and β-arrestin-2^-/- ^mice were examined using antibody that recognizes both β-arrestin-1 and 2 (preferentially reacts with β-arrestin-1). Epidydimal fat pads from male wild type (wt) and β-arrestin-2-/- (**B**) or wt and β-arrestin-1-/- (**C**) mice were harvested and treated with or without 2fAP for 0-120 minutes, and phosphor-AMPK determined as described in Figure 1. A total of 15 mice from each experimental group were analyzed. Representative westerns are shown in B and C. Levels of pAMPK normalized to tAMPK were determined as described in Figure 1 and mean   μ SEM pAMPK/tAMPK across all samples is presented as a bar graph of for all mice (**D**). The same data is also presented as dot plots showing fold changes over baseline in pAMPK levels for individual mice (**E**, note 0 minutes = baseline and is not included in the plot) and showing normalized phospho-AMPK levels of individual mice (**F**) *Statistically significant differences in mean pAMPK compared with wt mice (p < .005, n = 15). Statistically significant differences between 2fAP-treated and baseline pAMPK were only observed in β-arrestin-2-/- mice (p = .001, n = 15). This experiment was repeated in liver samples, included as Supplemental Figure 1. **G, H**. AMPK activity was determined as described in Figure 1 for AMPK immunoprecipitated from wt and β-arrestin-2^-/- ^fat and is depicted as fold change over baseline (**G**) and pmoles of ATP incorporated per mg of immunoprecipitated AMPK (**H**). **I**. Fat from β-arrestin-2^-/- ^mice was prepared as described in **A**, but was pretreated with STO-609 prior to activation of PAR_2 _with 2fAP for 0, 5 and 120 minutes. Shown are representative westerns of phospho-AMPK and total AMPK (upper panel) and a bar graph of normalized pAMPK levels in the presence and absence of STO-609 at each time point(lower panel).

**Figure 6:**
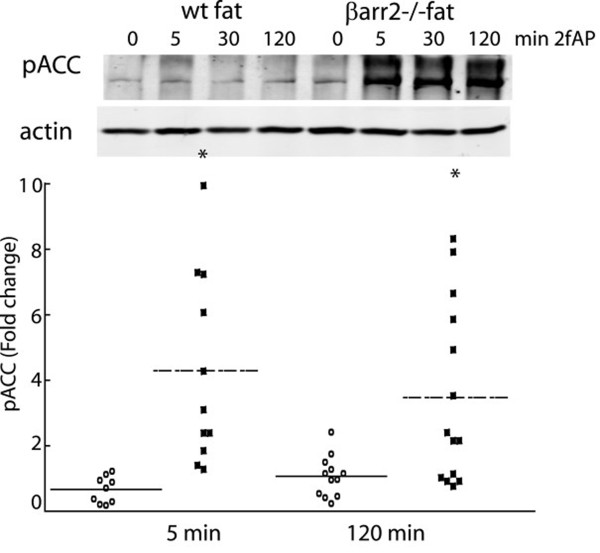
**PAR_2 _increases ACC phosphorylation in primary fat from β-arrestin-2^-/- ^mice**. Fat from wt and β-arrestin-2^-/- ^mice, prepared as described in Figure 4 was analyzed by western blot for pACC (upper panel) or actin (lower panel). Dot-plots showing normalized fold changes relative to baseline in pACC for individual mice are shown below (Baseline (untreated) is equal to 1, and is not shown in the graph). *Statistically significant changes in mean pACC compared with wt mice (p < .001, n = 11).

To confirm the inhibitory role of β-arrestin-2 on AMPK phosphorylation in primary cells, we investigated PAR_2-_stimulated AMPK phosphorylation in adipose tissue from wild type and either β-arrestin-1^-/- ^or β-arrestin-2^-/- ^mice. AMPK activity in adipose plays a key role in modulating the metabolic state of the animal [30,31] and we observe high levels of β-arrestin expression in primary fat as well as differentiated 3T3L1 adipocytes (Wang and DeFea, unpublished observations). Isolated epididymal fat was incubated with or without 2fAP for 5 and 120 minutes, then homogenized and analyzed by SDS-PAGE followed by western blotting for phospho-AMPK. In wt and β-arr1^-/- ^fat, no significant increase in AMPK activity was observed in response to PAR_2 _activation. However, in β-arr2^-/- ^fat, PAR_2 _promoted a 5-fold increase in AMPK phosphorylation (Figure 5B-E), and a 1.5-2.5-fold increase in AMPK activity (Figure 5F, G). Similar results were observed in liver from wt, β-arr-1^-/- ^and β-arr2^-/- ^animals (not shown). Pretreatment with STO-609 abolished PAR_2_-stimulated AMPK phosphorylation in β-arr2^-/- ^fat (Figure 5H, I), suggesting that AMPK phosphorylation by CAMKKβ is inhibited by β-arrestin-2. Consistent with these observations, PAR_2 _-stimulated phosphorylation of the AMPK substrate, ACC, was only observed in β-arr2^-/- ^mice (Figure 6). We conclude that PAR_2 _can promote CAMKKβ-dependent AMPK activation in primary fat, but under normal conditions this activity is suppressed by an inhibitory PAR_2 _pathway through β-arrestin-2.

### AMPK and CAMKKβ associate with β-arrestin-2

**Figure 7:**
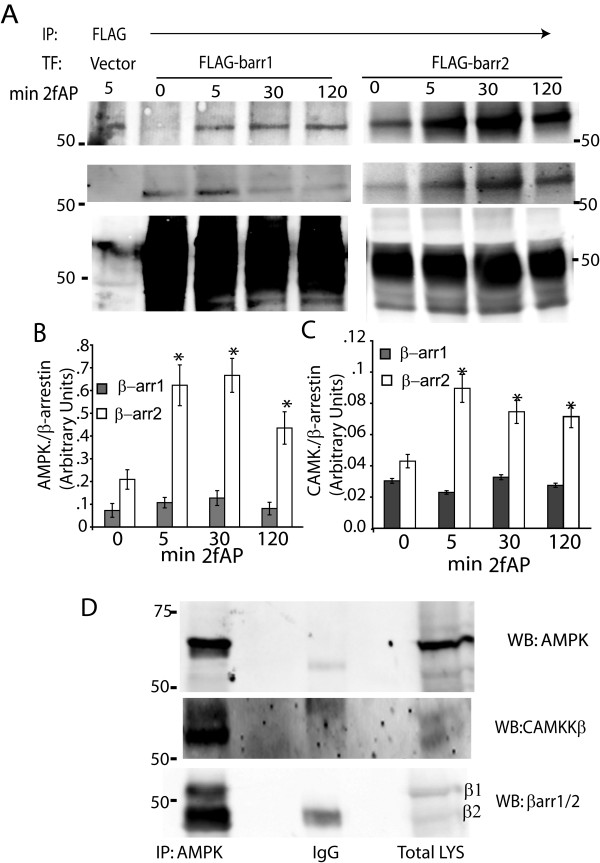
**Co-immunoprecipitation of β-arrestin-2, CAMKKβ and AMPK in NIH3T3 cells and primary fat tissue**. **A-C**. NIH3T3 cells, transfected with empty vector (as a negative control for Co-IPs) or flag-tagged β-arrestin-1 or 2, were treated with 2fAP for 0-120 minutes, lysed and β-arrestins immunoprecipitated with anti-flag, followed by western blotting with anti-AMPK, CAMKKβ and Flag. Expression of β-arrestin-1 was higher than that of β-arrestin-2 so AMPK and CAMKKβ protein levels in each immunoprecipitation were normalized to normalized to β-arrestin-1 or 2 levels. Bar graphs depicting quantification of immunoprecipitated AMPK (**B**) and CAMKKβ (**C**) are shown (n = 4). **D**. Epidydimal fat from wt mice was harvested and lysates were immunoprecipitated with AMPK or IgG followed by western blotting with antibody to AMPK (upper), CAMKKβ (middle panel) and β-arrestin-1/2 (lower panel). IgG heavy chain can be seen at 50kD in the β-arrestin panel. The band above IgG corresponds to β-arrestin-1 and the band below corresponds to β-arrestin-1.

Our studies on PI3K suggest that β-arrestins can form inhibitory scaffolds leading to decreased kinase activity. To examine whether β-arrestins similarly associate with AMPK and CAMKKβ, we performed co-immunoprecipitations in NIH3T3 cells transfected with flag-tagged β-arrestin-1 or β-arrestin-2, treated with and without 2fAP. Both AMPK and CAMKKβ could be co-precipitated with β-arrestin-2 and to a lesser extent, β-arrestin-1. Only β-arrestin-2 increased association with AMPK and CAMKKβ upon PAR_2 _activation (Figure 7A-C). Furthermore, a greater amount of both proteins associated with β-arrestin-2 relative to its expression level, than with β-arrestin-1 (Figure 7B, C). Confirming the specificity of this interaction, CAMKKβ and AMPK were not immunoprecipitated with anti-flag in mock transfected cells (see Figure 7A, left most lanes). We confirmed association of endogenous β-arrestins with AMPK and CAMKKβ in fat explants, where we immunoprecipitated AMPKα1 and probed western blots with β-arrestin-1/2 or CAMKKβ antibodies. AMPK could be co-immunoprecipitated with CAMKKβ and β-arrestin-2 (Figure 7D). Therefore, we conclude that β-arrestin-2 might form an inhibitory complex with AMPK and its upstream kinase, CAMKKβ.

### β-arrestin-2 directly inhibits CAMKKβ activity in vitro

**Figure 8:**
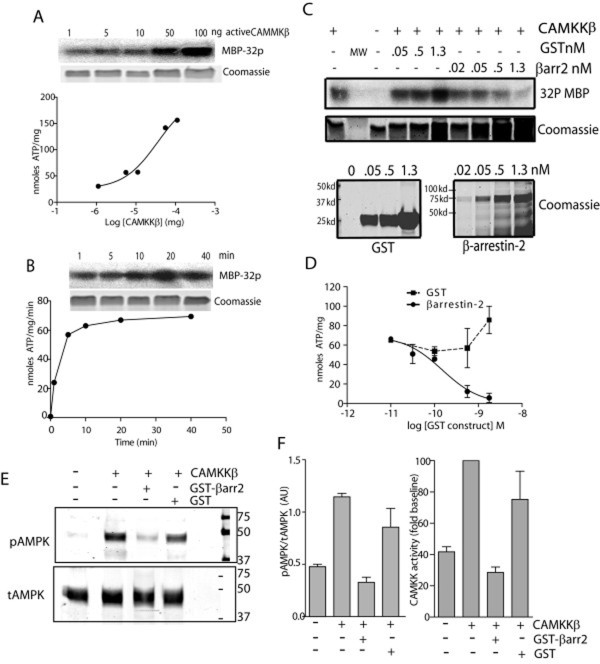
**β-arrestin-2 inhibits CAMKKβ activity and AMPK phosphorylation in vitro**. **A, B**. Conditions for optimal CAMKKβ concentrations and reaction times were determined. **A**. Increasing concentrations (0-100ng) of recombinant CAMKKβ were incubated with 1  μg of the substrate MBP in the presence of 32-P-ATP for 15 minutes. Reactions were analyzed by SDS-PAGE and radiolabel incorporation into excised MBP bands was determined. The upper panel is a representative autorad and the lower panel is the same gel stained with Coomassie Blue to show protein loading. **B**. Recombinant CAMKKβ (50ng) was incubated with 1  μg MBP and 32-P-ATP for 0-40 minutes and analyzed as described above. **C-D**. CAMKKβ kinase reactions using MBP as a substrate were repeated (50ng CAMKKβ, 15 minute reactions) in the presence of increasing concentrations of either recombinant GST or β-arrestin-GST and ^32^P-ATP. **C**. Representative autorad (Upper) of kinase reaction and corresponding coomassie stained gels (lower) are shown in the upper two panels. Levels of GST and β-arrestin-2-GST are shown in the inset at the bottom. **D**. Mean  μ SEM CAMKKβ activity in the presence of GST and GST-β-arrestin-2 is shown (n = 4). **E**. Recombinant AMPK was incubated with or without CAMKKβ alone, and with CAMKKβ in the presence of 500pM GST or 500pM GST-β-arrestin-2. Samples were analyzed by SDS-PAGE followed by western blotting with anti-phospho-AMPK (upper) and anti-total AMPK (lower) **F**. Graphs depicting normalized pAMPK levels (left panel) and fold increase in pAMPK relative to baseline (right panel). Baseline is defined as AMPK phosphorylation observed in the absence of CAMKKβ, n = 3.

To examine whether β-arrestin-2 can directly inhibit CAMKKβ activity, thus preventing phosphorylation of AMPK, we incubated recombinant GST-tagged β-arrestin-2 or GST alone (as a negative control) with recombinant CAMKKβ in the presence of ^32^P-ATP and the substrate myelin basic protein (MBP). CAMKKβ activity was determined by quantifying incorporation of ^32^P into MBP. Reactions were performed with 50ng CAMKKβ and carried out for 15 minutes, which resulted in maximal MBP phosphorylation (Figure 8A, B). Phosphorylation of MBP by CAMKKβ was inhibited in a dose-dependent fashion upon addition of β-arrestin-2-GST but not GST alone (Figure 8C, D), suggesting an overall inhibitory effect of β-arrestin-2 on CAMKKβ activity. We then specifically examined phosphorylation of AMPK on Thr172. CAMKKβ was incubated with recombinant heterotrimeric AMPK in the presence and absence of 500pM GST-β-arrestin-2 (the concentration at which MBP phosphorylation was inhibited by 80% in Figure 8D) or with GST alone, and phosphorylation determined by western blot using anti-phospho-AMPK and anti- total AMPK. CAMKKβ-stimulated AMPK phosphorylation was abolished by addition of recombinant GST-β-arrestin-2, but not GST (Figure 8E, F).

## Discussion

**Figure 9:**
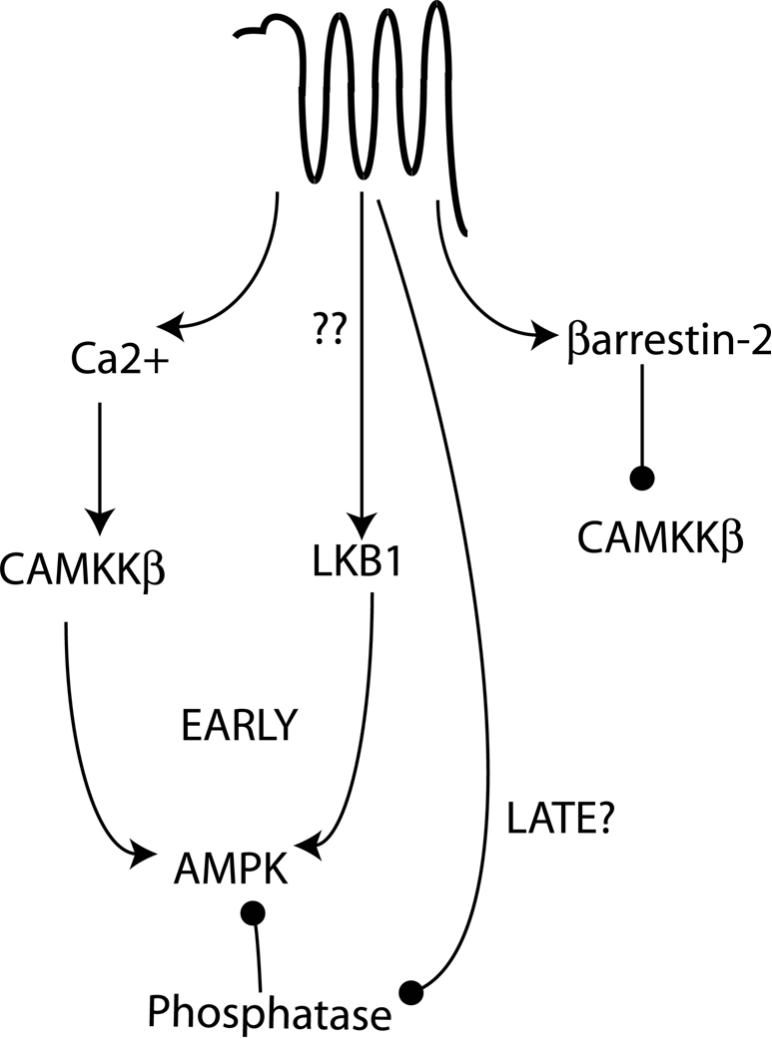
**Model for PAR_2 _regulation of AMPK activity**. PAR_2 _can activate G-protein pathways leading to Ca2+ mobilization and CAMKKβ activation, which then lead to phosphorylation and activation of AMPK. LKB1 also contributes to PAR_2-_stimulated AMPK activation, either through activation of the enzyme itself or by inducing a conformational change in AMPK that renders it more sensitive to phosphorylation by LKB1. At later time points, we predict that PAR2 may inhibit the activity of a phosphatase or protect AMPK from dephosphorylation, thus leading to the observed prolonged activation. Simultaneously, PAR_2 _can recruit β-arrestin-2 which binds both AMPK and CAMKKβ and prevents phosphorylation of AMPK. The resulting shift in the balance of phosphorylation and dephosphorylation of AMPK can lead to an observed decrease in overall AMPK phosphorylation in the presence of β-arrestin-2.

Here we describe a novel role for β-arrestin-2 in the regulation of AMPK, downstream of PAR_2_. We demonstrate that PAR_2 _can activate AMPK in the presence of low β-arrestin-2 levels, and inhibit it in cells with high levels of β-arrestin-2. While previous studies have investigated the mechanism of AMPK activation by another proteinase-activated receptor, PAR_1_, those studies did not deal with β-arrestins. Furthermore, the role of β-arrestins in signaling by the two receptors is quite different. PAR_2 _activation of AMPK involves the Ca^2+ ^sensitive enzyme, CAMKKβ, while the inhibitory pathway involves β-arrestin-dependent suppression of this same activity (depicted in Figure 9). As was observed for PAR_1_, LKB-1 may also play a role in PAR_2_-stimulated AMPK activation, but the sensitivity of this enzyme to β-arrestin-dependent regulation remains to be investigated. Research by ours and other groups over the last few years has revealed that β-arrestins can direct signals that oppose, facilitate, or act independently of a number of G-protein-directed signals[32]. With respect to PAR_2_, we have shown that Ca^2+ ^mobilization, downstream of Gαq activation, promotes nuclear MAPK activity, PI3K activity and LIMK activation, while β-arrestins promote inhibition of PI3K and LIMK and membrane sequestration of MAPK activity [1-4,33]. The predominance of one pathway over the other depends largely on the relative amounts of Gαq and β-arrestins, which appear to vary in a cell-type specific fashion [2]. Thus, the same extracellular signal can elicit distinct responses through the same receptor depending on the cellular context.

These findings also provide novel insight into the scaffolding functions of β-arrestin-2. To date, numerous binding partners have been identified for β-arrestins encompassing a diverse array of proteins including MAPKs, phosphatidylinositol kinases, actin assembly proteins, transcription factors, RhoGTPases, and ubiquitin ligases [8,32]. Interestingly, individual receptors promote recruitment of only a select group of these potential binding partners to β-arrestins. Part of this diversity can be explained by discrete domains within β-arrestins that serve as docking sites for different binding partners. Here we identify two new targets of β-arrestin-2-dependent scaffolding: CAMKKβ and AMPK which co-immunoprecipitate in cultured cells and in vivo. Although it is not yet clear whether either or both CAMKKβ and AMPK directly contact β-arrestin-2, it is likely that CAMKKβ directly interacts with β-arrestin-2, since addition of β-arrestin-2 blocked phosphorylation of both a non-specific substrate (MBP) and a specific one (AMPK1α). Furthermore, it is formally possible that AMPKα may directly bind β-arrestin, because it contains a stretch of amino acids within its N-terminus that bears with similarity to a recently identified conserved region in Jnk3 and CAMKγ, both of which constitutively bind β-arrestin-2 [34,35]. It will be interesting to determine whether AMPKα directly binds β-arrestin-2, whether it binds to the same region as Jnk3 and CAMKγ and whether these proteins compete for interaction with β-arrestin-2. While we demonstrated that interaction of β-arrestin-2 with AMPK and CAMKKβ in cells was enhanced by activation of PAR_2_, co-immunoprecipitation of all three proteins was observed in mouse fat in the absence of treatment, suggesting that this scaffolding complex may exist constitutively in vivo. Our data suggest that association of β-arrestin-2 with these proteins is strengthened by PAR_2 _activation. The conformational rearrangement that β-arrestin-2 undergoes upon receptor binding may alter the nature of the contacts between these proteins resulting in the observed inhibitory effect. Additional factors may also contribute to the inhibitory effect of β-arrestin-2 on AMPK in vivo. For example, β-arrestin-2 has been shown to bind and inhibit calmodulin which could contribute to the inhibition of CAMKKβ activity in cells. β-arrestin-2 has also been shown to scaffold PP2A to one of its substrates and scaffolding of PP2A to AMPK might further inhibit its phosphorylation[36]. Finally, β-arrestins also play a role in the desensitization of numerous receptors, ones that both activate and inactivate AMPK, such as adiponectin receptor. Thus, the absence of β-arrestin-2 may have the opposite effect on receptors that regulate AMPK independent of CAMKKβ.

These findings are likely to have implications in terms of metabolic regulation, although this possibility was not specifically addressed here. There is increasing evidence that obesity can be viewed as an inflammatory disorder, associated with increased circulating inflammatory cytokines and macrophage infiltration into fat, which in turn exacerbates defects associated with Type 2 Diabetes. PAR_2 _has been implicated in numerous inflammatory pathways and there is some evidence that β-arrestin levels can be altered under different physiological conditions [37,38] and in a mouse model of insulin resistance (db/db mice)[39]. β-arrestins have also been reported to contribute to insulin resistance by mediating a TNFα-induced inflammatory pathway[40]. There are a number of potential physiologically relevant agonists of PAR_2 _in the tissues examined here. Adipocytes secrete a trypsin-like enzyme called adipsin[41] that might activate PAR_2 _and Diabetes is associated with increased levels of mast-cell infiltration into the fat, and increased release of tryptase, another physiological activator of PAR_2_[42]. Factor VIIa, another known PAR_2 _agonist, is also reported to be elevated in Diabetes and decreased with strenuous exercise[43,44]. Future studies should address whether PAR_2 _activation has different effects on parameters associated with obesity in wild type versus β-arrestin-2 knockout mice, and address the effects of PAR_2 _on fat synthesis in cells.

## Conclusions

PAR_2 _can both activate and inhibit AMPK through distinct signaling pathways. First, via activation of CAMKKβ and to a lesser extent LKB-1, PAR_2 _can promote phosphorylation of AMPK and subsequent phosphorylation of its downstream substrate ACC. Second, via coupling to β-arrestin-2, PAR_2 _can inhibit AMPK phosphorylation. This inhibitory effect is mediated by association of β-arrestin-2 with AMPK and CAMKKβ, which results in direct inhibition of CAMKKβ activity.

## Methods

### Materials

All chemicals were from Sigma or Fisher Scientific except as otherwise indicated. PAR_2 _agonist, 2-Furoyl-LIGRL-O-NH_2 _(2f-AP), was synthesized by Genemed Inc. STO-609, a specific inhibitor for CAMKKβ was from Tocris.

### Animals

All procedures in the animal experiments were in accordance with the guidelines on the use and care of laboratory animals set by NIH and approved by the IACUC, University of California, Riverside. β-arrestin1^-/- ^and β-arrestin2^-/- ^in a C57BL/6 background were kindly provided by Dr. Robert Lefkowitz (DUMC, Durham, NC) and wild type C57BL/6 mice were from Jackson Labs. All strains of mice were bred at UC Riverside, were provided with standard rodent chow and water, and were housed under normal laboratory conditions (12 hr light/12 hr dark cycle). Age matched male mice (12~16-week-old) were used for this study.

### Cell Culture and Transient Transfections

Mouse embryonic fibroblasts (MEF) from wild type and β-arrestin knockout mice (from Dr. Robert Lefkowitz, DUMC) and NIH3T3 cells were grown in Dulbecco's modified Eagle's medium (DMEM, Mediatech) supplemented with 10% cosmic calf serum (Hyclone Laboratories) and maintained at 37°C with 5% CO_2_. Cells (approximately 70% confluent) were transiently transfected with 10  μg of FLAG-tagged β-arrestin-1 or -2 (from Dr. Robert Lefkowitz, DUMC) using Ca^2+ ^phosphate precipitation and harvested for experiments 36-48 h after transfection.

### Preparation of tissue and cell extracts

Visceral epidydimal fat pads and livers were harvested, washed in ice-cold saline and dissected rapidly into 3-5 mg pieces, followed by pre-incubation for 20 minutes in Krebs Ringer HEPES Buffer (pH 7.4) at 95%O_2_/5%CO_2 _at 37°C[45,46]. Samples were maintained at 95%O_2_/5%CO_2 _at 37°C for the duration of the experiments. Samples from individual mice were each assayed as separate experimental groups so that a given PAR_2 _stimulated AMPK response could be attributed to a single mouse. For cell culture studies, cells were used at 80% confluence and complete media was exchanged for serum free media 2 hours prior to the experiments. Tissues or cells were treated with or without 100nM of 2fAP- at 37°C and then homogenized in ice-cold lysis buffer containing TBS pH 7.4 supplemented with 1 mM EGTA, with proteinase and phosphatase inhibitors and either 1%Triton X-100 and 0.1% SDS (for total lysates analysis) or 1% NP-40 (for co-immunoprecipitations), followed by centrifugation for 10 min at 14,000 rpm at 4°C.

### Adenine Nucleotide Measurement by LC-MS/MS

NIH3T3 cells (grows at 80% confluence) were incubated with 100 nM of 2f-AP for 0-2 hrs, washed in cold PBS and 5% (wt/vol) of perchloric acid was added to the cells. Acid-insoluble material was removed by centrifugation, and perchloric acid was extracted from the supernatant by three washes with 10% excess (by volume) of a 1:1 mixture of tri-n-octylamine and 1,1,2-trichlorotrifluoroethane. The nucleotide mixture was subjected to online LC-ESI-MS/MS (liquid chromatography-electrospray ionization-tandem mass spectrometry) analysis using an Agilent 1100 capillary HPLC pump (Agilent Technologies) interfaced with an LCQ Deca XP ion-trap mass spectrometer (Thermo Fisher Scientific). The mass spectrometer was set up for monitoring the fragmentation of the [M-H]^- ^ions of AMP (m/z 346) and ATP (m/z 506). A 0.5 × 250 mm Zorbax SB-C18 column (5  μm in particle size, Agilent Technologies) was used, and the flow rate was 8.0  μL/min. A 5-min gradient of 0-20% methanol in 400 mM 1,1,1,3,3,3-hexafluoro-2-propanol (HFIP, pH adjusted to 7.0 by addition of triethylamine), followed by a 25-min gradient of 20-50% methanol in 400 mM HFIP was employed for the separation. The ratio of AMP over ATP was calculated by comparing the integrated areas of AMP to ATP from selected ion chromatograms (SICs) with the consideration of the differences in ionization and fragmentation efficiencies of the two nucleotides.

### Protein analysis and immunoblotting

Lysates from cells and liver or fat tissue (10 μg protein) were subjected to 8% SDS-PAGE gel and transfer to PVDFfl (Millipore) membranes, followed by Western blotting with the following antibodies: Rabbit polyclonal anti-phosphor-AMPK-Thr^172 ^or anti-phosphor-ACC-Ser^79 ^(both from Cell Signaling, 1:1000); mouse monoclonal anti-AMPKα1+2 (ProSci, 1:1000), rabbit anti-Flag (1:1000), rabbit anti-β-arrestin (A1CT, from Dr. Robert Lefkowitz, DUMC, 1:500) or rabbit anti-CAMKKβ (Upstate Biotech). Blots were imaged with Alexa^680^-conjugated rabbit (Invitrogen) and IR^800^-conjugated mouse (Rockland) secondary antibodies (1:40,000 each) using the LICOR Odyssey imaging system, and LICOR software was used to calculate integrated intensities of bands; phospho-AMPK and ACC levels were normalized total AMPK and actin respectively. Fold increases in phosphorylation were determined by dividing the band density observed with treatment by that observed in the absence of treatment. Images of Western blots were assembled using Adobe Photoshop 6.0 and imported into Adobe Illustrator. Some gels were spliced to eliminate blank lanes or lanes containing samples unrelated to the figure and splicing is indicated by a white space.

### Co-immunoprecipitation

Cleared lysates (500 μg of protein) were incubated with 20  μl of mouse anti-FLAG agarose conjugated antibodies pre-bound to protein A-agarose with mouse anti- AMPKα1 and 2 (1 μg/200 μg protein) coupled to Protein G agarose for 2hrs at 4°C on a rotator. Immune complexes were resolved by 10% SDS-PAGE and western blotting performed as described.

### In vitro AMPK Assays

AMPK was immunoprecipitated from cleared lysates with anti-AMPKα1/2 (1 μg/200  μg protein) as described above. Washed immune complexes were then used for AMPK assays. AMPK activity was determined by the incorporation of ^32^P-ATP (Perkin Elmer) into a synthetic substrate of AMPK, SAMS (HMRSAMSGLHLVKRR) peptide (Upstate Technologies), in the presence of 5 mM MgCl_2, _200  μM AMP and 200  μM [γ-^32^P] ATP (0.05  μCi/μl). Phosphorylated SAMS peptide was captured on phospho-cellulose strips (Pierce) and counted in a Beckman Scintillation counter; levels of AMPK present in each reaction was determined by western blotting of AMPK immune complexes after removal of reaction mixture, by comparing band density to that of a known quantity of purified recombinant AMPKα. Either the fold increase in activity was determined by dividing the normalized cpm incorporated with 2fAP treatment by that observed in the absence of stimulus or the moles ATP incorporated into each reaction was determined and expressed as nmoles ATP/mg enzyme/min.

### In vitro CAMKKβ Kinase Assays

GST alone and GST-tagged β-arrestin-2 (from Dr. Kerri Mowen, The Scripps Research Institute) was purified as described previously [3]. Recombinant active CAMKK2 (50ng, SignalChem) was incubated with the substrate MBP (5  μg/reaction), 200  μM ATP (with 0.5 μCI [γ-^32^P] ATP) and 5 mM MgCl_2 _in the presence of increasing concentrations of recombinant GST alone or GST-β-arrestin-2 at 30°C for 15 min. The enzyme concentration chosen represented the IC50 value determine in Figure 8A and the reaction time was chosen because at this point MBP phosphorylation was maximal (Figure 8B). Reactions were stopped with addition of Laemmli sample buffer and boiling; samples were then analyzed by SDS-PAGE followed by autoradiography. MBP bands were excised and phosphate incorporation was determined using a BECKMAN scintillation counter. For non-radioactive experiments, recombinant active CAMKKβ (50ng) was incubated with 200nG AMPK (Cell Signaling) in the presence of GST or purified β-arrestin-2-GST in PBS, 1 mM ATP and 5 mM MgCl2 at 30°C for 30 minutes. Reactions were analyzed by SDS-PAGE followed by western blotting with anti-phospho and anti-total AMPK antibodies.

### Data Analyses

All experiments were repeated a minimum of three times and results are presented as mean  μ S.E.M. Differences between multiple groups were examined by two-way ANOVA and Tukey *t*-tests using graphing software Microsoft Excel or GraphPad Prism, with *P *< 0.05 considered significant.

## Authors' contributions

**PW **performed all AMPK phosphorylation experiments and AMPK activity assays, in vitro CAMKK assays and co-immunoprecipitations and wrote the 1^st ^draft of the manuscript.

**YJ **and **YW **performed HPLC and Mass spectrometric analysis of nucleotides and provided the data shown in Figure 3.

**JS **assisted in the initial set up of AMPK assays in the laboratory.

**KD **designed the majority of the experiments, performed data analysis and was the senior investigator on this project.

All authors read and approve the final draft.
